# Modelling impacts of climate change and anthropogenic activities on inflows and sediment loads of wetlands: case study of the Anzali wetland

**DOI:** 10.1038/s41598-023-32343-8

**Published:** 2023-04-03

**Authors:** Mehran Mahdian, Majid Hosseinzadeh, Seyed Mostafa Siadatmousavi, Zohreh Chalipa, Majid Delavar, Ming Guo, Soroush Abolfathi, Roohollah Noori

**Affiliations:** 1 School of Civil Engineering, Iran University of Science and Technology, Narmak, Tehran, 1684613114 Iran; 2 Department of Water Engineering and Management, Tarbiat Modares University, Tehran, 14115-111 Iran; 3 School of Engineering, University of Warwick, Coventry, CV4 7AL UK; 4 Graduate Faculty of Environment, University of Tehran, Tehran, 1417853111 Iran; 5 Faculty of Governance, University of Tehran, Tehran, 1439814151 Iran

**Keywords:** Environmental sciences, Hydrology

## Abstract

Understanding the effects of climate change and anthropogenic activities on the hydrogeomorpholgical parameters in wetlands ecosystems is vital for designing effective environmental protection and control protocols for these natural capitals. This study develops methodological approach to model the streamflow and sediment inputs to wetlands under the combined effects of climate and land use / land cover (LULC) changes using the Soil and Water Assessment Tool (SWAT). The precipitation and temperature data from General Circulation Models (GCMs) for different Shared Socio-economic Pathway (SSP) scenarios (i.e., SSP1-2.6, SSP2-4.5, and SSP5-8.5) are downscaled and bias-corrected with Euclidean distance method and quantile delta mapping (QDM) for the case of the Anzali wetland watershed (AWW) in Iran. The Land Change Modeler (LCM) is adopted to project the future LULC at the AWW. The results indicate that the precipitation and air temperature across the AWW will decrease and increase, respectively, under the SSP1-2.6, SSP2-4.5, and SSP5-8.5 scenarios. Streamflow and sediment loads will reduce under the sole influence of SSP2-4.5 and SSP5-8.5 climate scenarios. An increase in sediment load and inflow was observed under the combined effects of climate and LULC changes, this is mainly due to the projected increased deforestation and urbanization across the AWW. The findings suggest that the densely vegetated regions, mainly located in the zones with steep slope, significantly prevents large sediment load and high streamflow input to the AWW. Under the combined effects of the climate and LULC changes, by 2100, the projected total sediment input to the wetland will reach 22.66, 20.83, and 19.93 million tons under SSP1-2.6, SSP2-4.5, and SSP5-8.5 scenarios, respectively. The results highlight that without any robust environmental interventions, the large sediment inputs will significantly degrade the Anzali wetland ecosystem and partly-fill the wetland basin, resulting in resigning the wetland from the Montreux record list and the Ramsar Convention on Wetlands of International Importance.

## Introduction

The Anzali wetland is located in the north of Iran and is connected to the Caspian Sea through a shipping channel. This exorheic wetland is characterized by rich biodiversity (Flora and Fauna), habitats for migratory birds, and important ecological functions^1^. The Anzali wetland is listed as a coastal waterbody under the Ramsar Convention on Wetlands of International Importance, and Important Bird and Biodiversity Area. The wetland’s water surface area is estimated to be 44 km^2^ in 2020, but the long-term monitoring data show that the water surface area and the effective volume of the wetland has been gradually shrinking during the last century. A study conducted by Japan International Cooperation Agency (JICA) shows that the wetland’s water surface area in 1989 (~ 52 km^2^) was approximately one-fifth of that in 1930 (~ 258 km^2^)^1^.

Degradation of the Anzali wetland is exacerbated by a combination of natural and anthropogenic stressors. Natural drought events and a decrease in the Caspian Sea’s mean water level contribute to the decline of wetland’s surface area^1^. In addition, anthropogenic activities in the Anzali wetland watershed (AWW), such as land use/land cover (LULC) changes and extensive agricultural practices, have led to inflow decline and increase in sediment inputs to the wetland through altering the watershed’s hydrological processes^1,2^. Given the severe degradations of the Anzali wetland, it was enlisted in the Montreux Record in 1993, a section of the Ramsar Convention^3^ that lists the wetlands specified with the changes in ecological character due to technological growth, pollution, or other anthropogenic interferences. The continued inflow declines and increase in sediment input to the wetland could collapse the wetland’s ecosystem and food chain, severely harm the habitat and biodiversity, create salt storms, change the microclimate, and introduce acute impacts on local agriculture and the regional public health^4^. Hence, a detailed understanding of the future impacts of climate and LULC changes on the inflow and sediment inputs to the wetlands is essential for protecting their ecosystem health and functions, as well as in-depth environmental risk assessment of wetlands^5^.

Utilizing Global Circulation Models (GCMs) projections of the future climate is a common approach for investigating climate change impacts on water resources^6–8^. However, GCMs have various structural initializations and the mathematical parameterizations of various physical processes, which vary between different GCMs. To overcome the inconsistency in GCMs, and reduce the uncertainty in the projected results, multi-GCMs techniques are adopted in hydro-climatological studies^7,9^. In addition to climate change, LULC changes can significantly influence hydrology and water quality of water bodies^10–16^. Given the nonlinear interaction of climate and LULC changes with the hydrological processes, a static LULC map of the watershed cannot provide accurate snapshot of the future conditions. This study, for the first time, investigates the combined effects of climate change and LULC to project streamflow and sediment input into the Anzali wetland, using the Soil and Water Assessment Tool (SWAT) model^17^. The Land Change Modeler (LCM)^18,19^ was used to predict future LULC maps across the AWW. Climate projections are produced using the Multi-GCMs via Coupled Model Intercomparison Project-6 (CMIP6)^20^ models, which can accurately map the future climate conditions of watersheds relative to their prior conditions^21^. The projected climate change and LULC changes are introduced to the SWAT model to analyze streamflow and sediment yields for the AWW. Anzali wetland’s watershed has been subjected to acute anthropogenic activities over the past decades, where the change in both climate change drivers and LULC is expected to continue in the future^1,2^. Therefore, the main contribution of this study is to investigate the combined effects of climate and LULC changes on the inflow rates and sediment loads to AWW using SWAT model. The uncertainties associated with the projected results are discussed and a methodological approach to quantify the effects of climate change and LULC for wetlands is proposed.

## Materials and methods

### Study area

**Figure 1:**
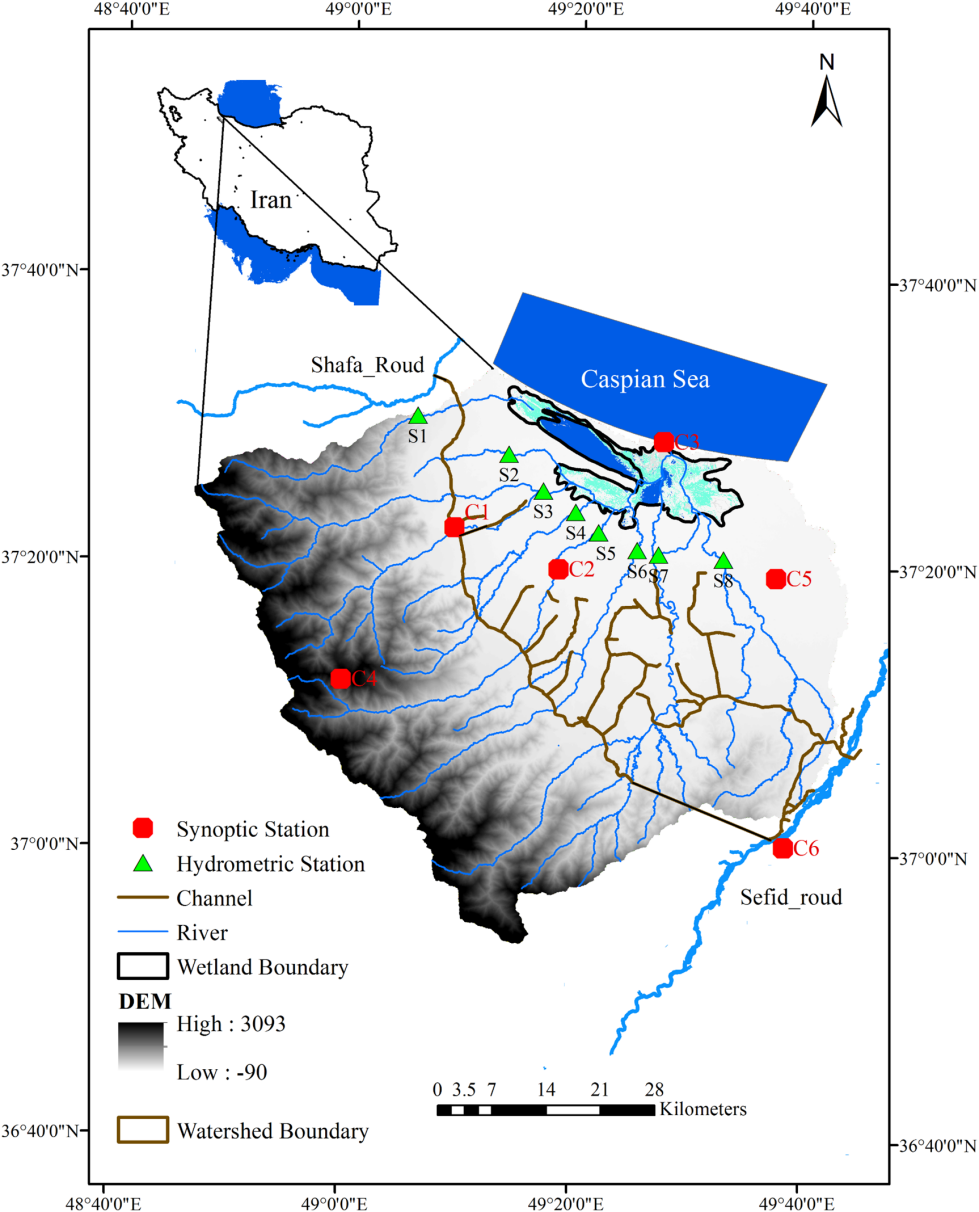
The Anzali wetland watershed (AWW) boundaries with the position of local rivers, hydrometric stations, synoptic stations, deviation channels, and digital elevation map (DEM) (This figure was created in the environment of QGIS, version 3.2).

**Figure 2:**
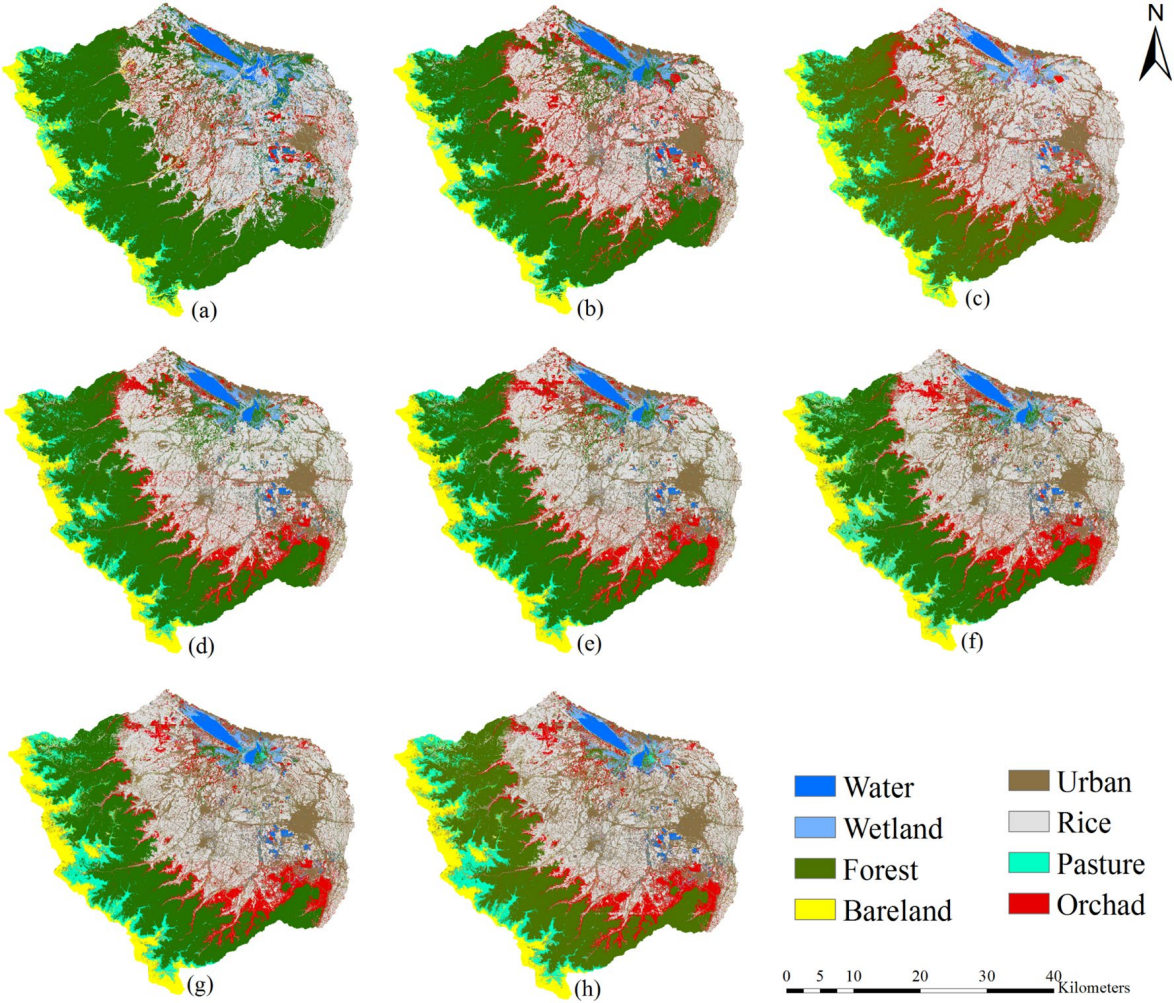
Satellite-based produced land use land cover (LULC) maps in (**a**) 2001, (**b**) 2014, and (**c**) 2020. Projected LULC maps using the Land Change Modeler (LCM) in (**d**) 2020, (**e**) 2040, (**f**) 2060, (**g**) 2080, and (**h**) 2100 (This figure was created in the environment of QGIS, version 3.2).

The Anzali wetland is a coastal lagoon located on the southwestern coast of the Caspian Sea in Iran, spanning from 48° 45' to 49° 42' E longitudinal and 36° 55' to 37° 32' N latitudinal (Fig. 1). The Anzali wetland is a large freshwater lagoon with the water surface area of ~ 44 km^2^, fed by nine rivers and streams. The AWW area is ~ 3600 km^2^ and consists of diverse terrain including mountainous areas in the south, sparse forests and flatlands in the north. The elevation across the watershed ranges from − 90 m in the north to 3318 m in the south. The Anzali wetland is of significant importance for the ecology and economy of the region and has various hydro-ecological functions, such as improving water quality and water purification, flood and erosion control, groundwater recharge, wildlife nursery, and biodiversity. The climate of the AWW is predominantly humid, with an average temperature of 8 °C and 23 °C, in synoptic stations in the coldest (i.e., January) and warmest (i.e., August) months, respectively. The diurnal temperature fluctuation across the AWW is minimal due to high average annual humidity of 66% and precipitation rate around 1185 mm. Monthly precipitation is the most abundant during the month of October and the least abundant in June. Nine main rivers are entering the wetland, including Chafroud (S1), Behambar (S2), Morghak-Khalkaei (S3), Palangvar (S4), Masouleh Roudkhan (S5), Shakhraz (S6), Pasikhan (S7), and Pirbazar (S8) (Fig. 2). The Anzali wetland has rich ecosystem in terms of biodiversity, Flora and Fauna, with about 150 species of birds (resident and migrant), 49 species of fish, 17 species of amphibians and reptiles, and 31 species of mammals. The flora species are mainly classified into Phragmites, submerged plants, and Azolla communities^1^.

### LULC extraction

Changes in LULC can strongly influence both streamflow and sediment yield at watershed scale^10,14^. Given that no existing LULC map for the AWW was available, this study develops LULC maps for the case study region using satellite imagery data. Landsat 7 Enhanced Thematic Mapper Plus (ETM +) images for the AWW were obtained for August 2001^22^. To understand and quantify the temporal changes in LULC across the AWW, Landsat 8 Operational Land Imager and Thermal Infrared Sensor (OLI/TIRS) images were used for August 2014 and 2020^22^. Satellite images were used for the cloud-free sky condition, and different vegetation types coverage across the watershed area was captured^23^. Prior to further processing of the LULC maps, FLAASH atmospheric correction^24^ and fusion algorithms^25^ were employed to correct atmospheric noises and increase the spatial resolution of the produced maps from 30 to 15 m. Feed-forward back-propagation neural network algorithm^26,27^ was used to classify the maps. The LULC maps were classified into eight major classes including forest, rice paddies, orchard, urban, bare land, wetland, pasture, and freshwater. Rice cultivation is flood irrigation, and orchard are rain-fed products. The classified LULC provided by JICA^1^, and the orchard farm maps developed by the Gilan Regional Water Authorities are adopted as reference maps to distinguish between the rice and orchard classes in the case study region. Thereafter, more than 50 points for each land-use class were extracted from Google Earth as true data^28^ to further assess the accuracy of the extracted land-use map using the Kappa coefficient^29^ and overall accuracy indices.

### LULC prediction

A wide range of natural and anthropogenic drivers could influence the LULC changes, which should be accurately considered in the LULC prediction. Following on previous studies^30–32^, the statistic variables of digital elevation model (DEM), slope, distance from the roads, proximity to rivers, distance from cities, distance from the wetland, and evidence likelihood are hypothesized to impact the LULC change in the AWW. Detailed descriptions and justifications about the selection of these variables are given in the Supplementary S1. Cramer's V index is used to examine each potential driver hypothesized to impact the LULC change in the AWW^33^.

The Land Change Modeler (LCM)^18,19^ is utilized to analyze the influence of the key anthropogenic and natural drivers on the future trends of LULC. An empirical approach for understanding the historical LULC changes and predicting the future transitions based on the explanatory variables is adopted in LULC modelling. The LCM simulation is initiated by developing a multi-layer perceptron neural network (MLPNN) to create the transition potential matrix based on the LULC maps in 2001 and 2014, the driving variables, and considering sub-models. Then, the LCM performs a series of empirically evaluated transition sub-models to analyze the changes and predict the future states. Each sub-model simulates the alteration from one LULC class to another^18,19^. The sub-models were assumed based on net change produced by the LCM, including conversion from wetland to rice paddies, forest to pasture and orchard, orchard to rice and urban, rice to urban, and pasture to bare land. Markov Chain analysis is carried out to determine the quantity of change for the selected prediction date. The transition potential maps and projected quantity of change for each transition are then used for LULC simulations with spatial allocation using a greedy selection algorithm.

The LULC map of the AWW in 2020 was extracted from a satellite image and used for validation of the LCM projected results. Four Kappa indices (K-indices) of agreement, i.e., indecesKno (Kappa for no information), Klocation (Kappa for location), Kstandard (Kappa for standard), and KlocationStrata (Kappa for stratum-level location) were determined to evaluate the performance of LCM^34,35^. The K-index greater than 0.8 indicate an acceptable agreement between the LCM projections and the actual LULC map^36^. Finally, the validated LCM was utilized to project the long-term evolutions of LULC maps in 2040, 2060, 2080, and 2100.

### Statistical climate data downscaling

GCMs have become more sophisticated and robust tools over the past decade, and their ability to simulate the global climates have significantly improved. However, the spatial resolution of GCMs remains relatively coarse, and their outputs have a considerable systematic bias relative to observational datasets. Statistical downscaling techniques have been proposed to bridge the gap between the resolution of global-scale climate models and the regional to local-scale processes and reduce the downscaling bias which can be a source of error for impact assessment studies^37^. In this study, the daily maximum air temperature (Tas_max_), daily minimum air temperature (Tas_min_), and daily cumulative precipitation were obtained for 12 GCMs (see Supplementary Table S1) based on the CMIP6^20^ from 1986 to 2100, which was downloaded from ESGS^38^.

The statistical downscaling was performed based on the Euclidean distance to assign each GCM grid point to the nearest synoptic stations. The quantile delta mapping (QDM) technique^39^ was used for bias correction of GCMs relative to the based observational data in each synoptic station (i.e., 1986–2014), executed by the "qmap" package in R (version 4.1.2). The QDM reduces systematic error embedded in GCMs raw data, avoids corruption in extreme climatological events, especially precipitation, and maintains model-projected relative changes in quantiles while correcting systematic biases in the quantiles of the modelled series regarding the observation data^39^.

The best GCMs that matched the observational data in synoptic stations were selected based on the bias correction results. Given the selected five GCMs, the hydrological response of the wetland watershed in terms of flow and sediment yields was simulated with SWAT under three future climate scenarios published by the Intergovernmental Panel on Climate Change^40^ (IPCC) on the Shared Socioeconomic Pathways (SSPs) scenarios, i.e. SSP1-2.6, SSP2-4.5, and SSP5-8.5. The combinations of SSPs with the five GCMs used in this study cover a wide range of climate variability scenarios. We used correlation coefficient (CC), normalized root mean square error (NRMSE), and Kolmogorov–Smirnov statistics (KS)^41,42^ to examine the performance of the GCMs for precipitation. The GCM results are well matched the observational air temperature data, and therefore, we only selected the CC to quantify the accuracy of GCMs for the maximum and minimum air temperature. It should be noted that in C1 and C4 synoptic stations, the air temperature data was not measured.

### Streamflow and sediment load modeling

SWAT (Soil and Water Assessment Tool) is conceptual and continuous-time model, which is used as a comprehensive semi-distributed river basin model to analyze surface water quality and quantity under various land management practices^17^. The governing hydrology model used in this study is based on the water balance equation (Eq. (1)):

1 $${SW}_{t }=SW+ \sum_{t=1}^{t}( {R}_{i}- {Q}_{i}-{ET}_{i}-{P}_{i}-{QP}_{i })$$

where, *SW*_*t*_ is the final soil water content [mm of H_2_O], *SW* is initial soil water content, *t* is time [day], and *R*, *Q*, *ET*, *P*, and *QR* are the daily amounts of precipitation, runoff, evapotranspiration, percolation, and return flow, respectively^17^. We used the Hargreaves^43^ and SCS curve number methods^44^ to calculate *ET* and *SW*, respectively.

The sediment yield transported to the surface runoff was computed using Modified Universal Soil Loss Equation (MUSLE)^45^ (Eq. (2)):

2 $$Sed=11.8 .{({Q}_{surf}.{q}_{peak}{.A}_{HRU})}^{0.56}.{K}_{USLE}.{C}_{USLE}{.P}_{USLE}.{LS}_{USLE}.CFRG$$

where *Sed* is the sediment yield on a given day [metric tons], *Q*_*surf*_ is the surface runoff volume, *q*_*peak*_ is the peak runoff rate, *A*_*HRU*_ is the area of the HRU [ha], ${K}_{USLE}$ is the USLE soil erodibility factor, ${C}_{USLE}$ is the USLE cover and management factor, ${P}_{USLE}$ is the USLE support practice factor, ${LS}_{USLE}$ is the USLE topographic factor, and *CFRG* is the coarse fragment factor.

The AWW was partitioned into 70 sub-basins using DEM derived from ASTER satellite data (GDEM V_2_). Each sub-basin is divided into hydrologic response units (HRUs), lumped land areas with a combination of a LULC map extracted in 2014, an FAO-based soil map^46^ and a three-level slope map (i.e. 0–15%, 15–30%, and 30–99%). Due to the high variability of the watershed elevation (− 90 to 3318 m), ten elevation bands were introduced to the SWAT model to comprehensively consider precipitation and temperature distribution. No threshold was defined for HRUs generation to avoid excluding the LULC classes, which occupy smaller areas in the AWW (e.g., urban class), resulting in the generation of 1679 HRUs.

Weather data for 1986–2020 period were received from the Iran Metrological Organization at six synoptic stations (Fig. 1). The agricultural operation information across the basin (e.g., crop cultivation season, water usage of rice, tillage, and harvest) was predominantly obtained from the Iran Water Resources Management Company (IWRMC), with inputs from the experts and local farmers. The data obtained shows the crop-growing season begins in early April and lasts until late September. Approximately 70% of the irrigation water required for rice cultivation, as the only irrigated crop, is supplied through the Sefidrood and Shafarood rivers, which are both, located outside of the watershed and enter the watershed by two deviation channels (Fig. 1). The remaining water needs for the crop is provided by local rivers across the AWW. The irrigation efficiency is estimated to be 40%. For simulation, the paddy field in the SWAT model pothole was defined in HRUs, covered by rice in the AWW. The net evapotranspiration of the paddy field is estimated to be 850 mm.

The daily discharge data for eight hydrometric stations, obtained through the IWRMC database from 1986 to 2020, were converted to monthly time series. Sediment rating curve^47^, which are better captured at monthly steps^48^, were tuned for each hydrometric station using at least 300 sediment samples. The measured monthly streamflow and sediment load data calculated by the tuned sediment rating curves were used to calibrate and validate SWAT model outputs. Calibration and validation of the SWAT model were carried out with the streamflow and sediment load data using an integrated algorithm of sequential uncertainty fitting Version-2 (SUFI-2) module in the SWAT-CUP^49^. The historical periods of 1986–1988, 1989–2012, and 2013–2020 were used for the model warm-up, calibration, and validation, respectively. Each period introduced for the SWAT calibration and validation process contains both dry and wet events, which can better represent the watershed condition in the model^50^.

To examine the performance of SWAT model, the Nash–Sutcliffe efficiency (NSE) index^51^ was selected as it can more accurately estimate the annual average water yield and related water balance components^52^ (e.g., surface runoff, evapotranspiration, lateral flow, and groundwater flow). The coefficient of determination (R^2^)^53^ is determined for the modelling results to further evaluate the model performance in estimating streamflow and sediment load across the AWW. Tuned models with NSE ≥ 0.5 and R^2^ ≥ 0.6 are supposed to have a satisfactory performance^54,55^.

Additionally, P-*factor* and R-*factor* were used to quantify SWAT model performance. The P-*factor* determines the percentage of the observed data falls into the 95 percent prediction uncertainty (95PPU) band. The R-*factor* is determined as the average thickness of the 95PPU divided by the measured data's standard deviation, providing insights into the model uncertainty. This study considers a P-*factor* ≥ 70% and R-*factor* ≤ 1.5 acceptable for river discharge. In terms of the sediment load, the reference P-*factor* greater than 50% (and even 40%) and the R-*factor* smaller than 2 (and even 3) can be acceptable^56^.

Calibration of the SWAT model was initiated by running both precipitations lapse rate (PLAPS) and temperature lapse rate (TLAPS) in SWAT-CUP, following the suggestions of Abbaspour et al.^57^, which separate driving factors from other parameters through the calibration process. Thereafter, several parameters, as suggested by others, were initially selected for streamflow calibration using the global sensitivity analysis method. This study uses the larger absolute value of *t*-stat, and the smaller *P*-value obtained through the global sensitivity analysis to determine the sensitive parameters^57^. Note that the upper lands in the AWW, which are covered mainly by forest and located in the steep zones, were separated from lowland in the flat zones in the calibration process of the SWAT model.

Downscaled climate data of five GCMs were fed to the tuned SWAT model for projecting sediment load and streamflow under three climate scenarios of SSP1-2.6, SSP2-4.5, and SSP5-8.5. The changes in sediment load and streamflow in the AWW were projected by considering the simultaneous impacts of climate and LULC changes. For this purpose, the Land-Use Update Tool^58^, was used to produce the HRU-fractions for the introduce map to the SWAT model, i.e., the extracted sattile based LULC maps in 2001, 2014, 2020, and the LCM projected LULC maps of 2040, 2060, 2080, and 2100. HRU-fractions were annually computed using a linear interpolation of LULC changes during the study period.

## Results and discussion

### LULC changes

Using the Landsat 7 (ETM +) and Landsat 8 (OLI/TIRS) remote sensing imageries, the LULC classification maps across the AWW were determined for the years 2001, 2014, and 2020. Different classes of LULC and their percentage area were identified and quantified (Table 1). Figure 2 depicts the spatial distribution of LULC maps for the AWW. Comparison of the data from 2001 to 2020 demonstrates intense LULC changes in forest and urban areas. The forest areas decreased by 10.5%, and urban areas increased by 7.2% across the AWW. Urbanization has increased in recent years mainly to accommodate for tourism attractions of the wetland, leading to converting rice and orchard farms to urban areas. Given that the central north regions of Iran are the main provider of rice for the country, the agricultural lands in this region cannot decrease significantly. As a result of this, the forests have been converted into agricultural lands to compensate for the urbanization across the AWW. Deforestation has also occurred in the upstream of the wetland due to excessive logging and grazing activities^1^. Other land use classes such as rice, orchard, bare land, and pasture have increased by 2.4%, 2.8%, 0.2%, 7.2%, 0.8%, respectively.

**Table 1:** Land use land cover (LULC) area percentage of satellite based remote sensing and the projected LULC maps obtained from the Land Change Modeler (LCM).

Year	2001	2014 (change)^a^	2020 (change)^a^	2020 LCM (change)^b^	2040 LCM (change)^a^	2060 LCM (change)^a^	2080 LCM (change)^a^	2100 LCM (change)^a^
LULC								
Water	2.1	1.9 (− 0.2)	1.3 (− 0.8)	1.8 (0.5)	1.8 (− 0.3)	1.8 (− 0.3)	1.8 (− 0.3)	1.8 (− 0.3)
Forest	47.2	39.6 (− 7.6)	36.7 (− 10.5)	35.4 (− 1.3)	32.9 (− 14.3)	30.7 (− 16.5)	29.3 (− 17.9)	28.9 (− 18.3)
Rice	25.6	26.6 (1)	28 (2.4)	29.7 (1.7)	28.7 (3.1)	28.1 (2.5)	27 (1.4)	26.8 (1.2)
Orchard	6.0	11.1 (5.1)	8.8 (2.8)	8.7 (− 0.1)	9.4 (3.4)	9.6 (3.6)	9.5 (3.5)	9.2 (3.2)
Bare land	5.3	5.6 (0.3)	5.5 (0.2)	6 (0.5)	6.4 (1.1)	6.7 (1.4)	6.7 (1.4)	6.8 (1.5)
Urban	5.1	9.2 (4.1)	12.3 (7.2)	12.4 (0.1)	14.6 (9.5)	16.1 (11)	17.2 (12.1)	18.1 (13)
Pasture	3.6	3.3 (− 0.3)	4.4 (0.8)	3.9 (− 0.5)	4.4 (0.8)	5.3 (1.7)	6.7 (3.1)	6.7 (3.1)
Wetland	5.0	2.8 (− 2.2)	3 (− 2)	2.2 (− 0.8)	1.9 (− 3.1)	1.9 (− 3.1)	1.7 (− 3.3)	1.7 (− 3.3)

^a^Change % relative to 2001.

^b^Change % relative to actual map of 2020.

Water coverage in the AWW has decreased due to the climate change impacts^59^ and fluctuations in the water level of the Caspian Sea^1^. Due to an increase in the regional and national nutritional needs, the fertile lands around the wetland have been gradually converted to agricultural lands. Separating rice from orchards is essential because these classes need different agricultural practices. Rice is considered a flood-irrigation crop, while orchards are rain fed areas. Without appropriate spatial separation between these agricultural activities, the water needs and consequently river discharge can be affected. Tillage, which influences sediment yield, only occurs for the rice crop. In addition, separation of bare land areas (without vegetation cover) and pasture zones (with a good vegetation cover), which are located on steep slopes in the AWW, could lead to more accurate results, especially for the sediment yield prediction. These important factors were not considered in the previous studies aimed to project the streamflow and sediment yield in the AWW^2,59^. The LCM results generated in this study reveal the Kappa coefficients of 0.89, 0.87, and 0.85 for the LULC maps in 2001, 2014, and 2020, respectively. Also, the overall accuracy of LULC maps was 0.9, 0.89, and 0.9 in 2001, 2014, and 2020, respectively. The statistical analysis highlights the robustness and appropriateness of LULC modelling strategy from the satellite remote sensing data^36^.

### Prediction of future LULC

The Cramer’s *V* values of drivers which have influenced the transition of the LULC classes between 2001 and 2014 are 0.21, 0.26, 0.27, 0.29, 0.36, 0.40, 0.61 for closeness to rivers, distance from cities, distance from roads, slope, distance from the wetland, DEM, and evidence likelihood, respectively. Given that the Cramer’s *V* values of more than 0.1 are deemed influential, all the investigated variables significantly contribute to the transition maps^33^. The closeness to rivers (Cramer’s *V* = 0.21) and the evidence likelihood (Cramer’s *V* = 0.61) are the least and most important drivers of the transition maps, respectively. The Kno, Klocation, KlocationStrata, and Kstandard indices of LULC generation through LCM in validation step were 0.85, 0.83, 0.81, and 0.80, respectively. The results of the *K*-index show acceptable performance of the LCM (> 0.8)^36^. The validation results and the changes in different classes of the LULC maps are shown in Table 1. The LCM best and worst predicting performance of LULC classes are observed for rice and urban classes, respectively.

Given the robust results of the tuned LCM, this study adopts the validated and calibrated LCM to predict the future LULC maps in 2040, 2060, 2080, and 2100 (Fig. 2). Relative changes in the different classes of the projected LULC maps to the LULC map obtained in 2001 (Table 1) show that the forest area decreases by 14.3%, 16.5%, 17.9%, and 18.3% in 2040, 2060, 2080, and 2100, respectively. The projection for urban areas shows increases by 9.5%, 11%, 12.1%, and 13% in 2040, 2060, 2080, and 2100, relative to urban area detected at 2001, respectively. In general, due to the natural and human-made impacts, the forest and urban areas are expected to fluctuate in the future, as such, the findings of this study is aligned with similar studies^30,32,60^. The results show that deforestation and urban expansion will continue until 2060 similar to those rates observed between 2001 to 2020. Both deforestation and urbanization trends are predicted to slow down after 2060. Based on the soft prediction map, which demonstrates pixel vulnerability to the change, the rest of the LULC classes would resist more relative to historical trends against the changes. On the other hand, the rice class across the AWW increase by 2.4% from 2001 to 2020 (Table 1). However, according to the LCM model's projection of the future land use and land cover (LULC) map, the rice crop area is expected to decrease from 2020 to 2100. This decline is mainly due to urbanization, which is driving changes in the flat areas surrounding the AWW. The LCM's soft prediction map indicates that the vulnerable areas of the rice crop are gradually being converted into urban areas, a trend that is also affecting the orchard class.

In the flat area across the AWW, the orchard class is being gradually converted to urban and rice areas, while in the slope area, it replaces the forest area. However, the area gained by the orchard class is less than the area lost across the AWW. Deforestation is the primary factor driving changes in the bare land and pasture area. When deforestation takes place in the southern part of the AWW, the forest area is initially converted to pasture, and with grazing activity, the pasture area is converted to the bare land class. Although in wet years, bare land can convert to pasture area across the AWW, the sub-models of the LCM model are restricted to eight sub-groups, which limits the consideration of this change. As a result, the bare land and pasture areas tend to have slight differences from their condition in the base period. The LCM results for the future LULC maps revealed that the temporal changes in the LULC classes are significant and can dominantly influence the hydrology and water quality processes at the watershed scale^2,10,14^. The results show that previous studies that excluded the changes in LULC in the AWW cannot accurately project the watershed streamflow^59^.

### Downscaling and projection of climate variables

**Figure 3:**
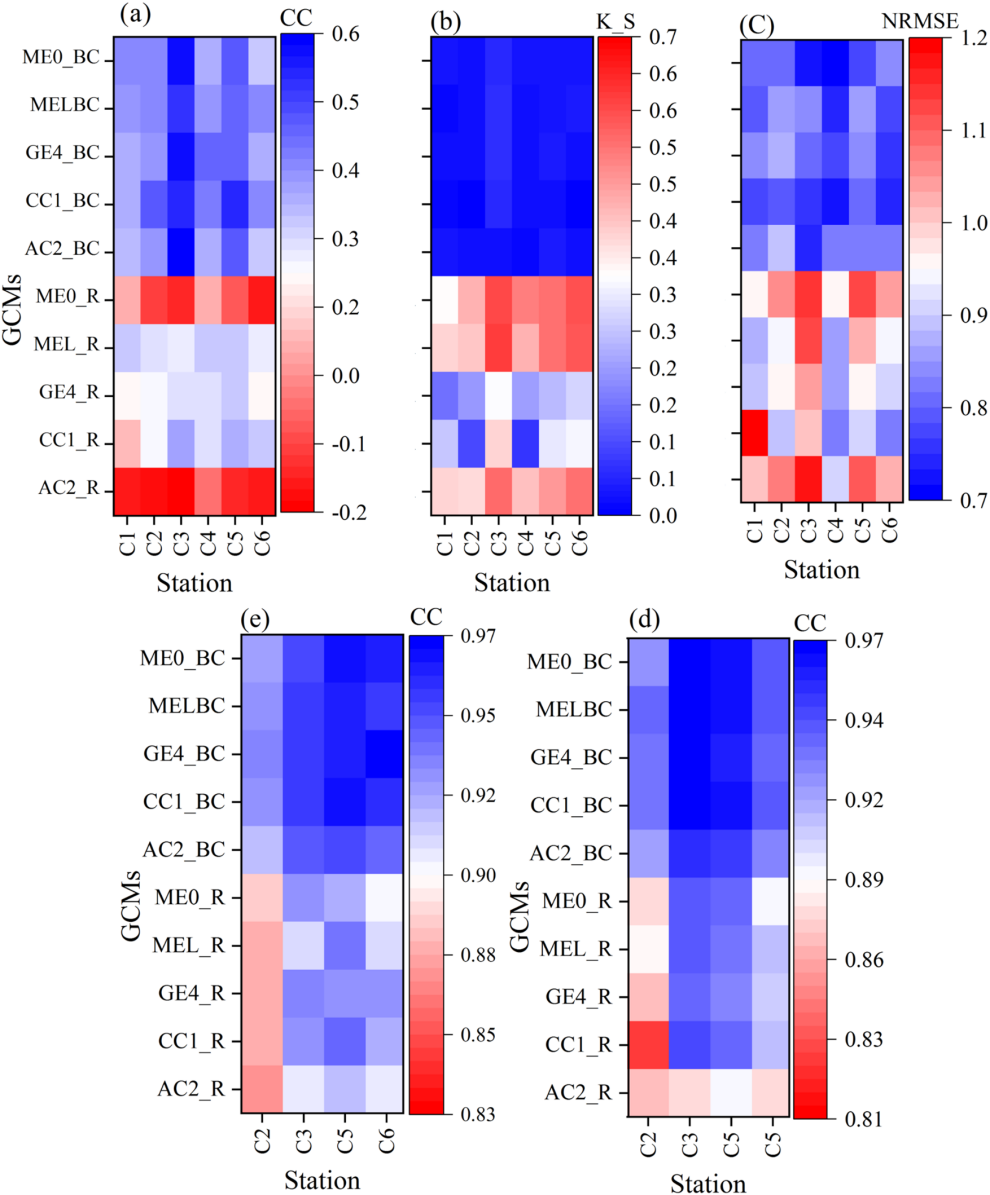
Summary of the statistical performance of raw General Circulation Model (GCM) (suffix _R), and bias-corrected GCM (suffix _BC), for monthly precipitation based on (**a**) the Kolmogorov–Smirnov statistics (KS), (**b**) correlation coefficient (CC)*,* and (**c**) normalized root mean square error (NRMSE). Summary statistics of the monthly performance of raw GCM (suffix _R) and bias-corrected GCM (suffix _BC) based on the CC for (**d**) minimum air temperature and (**e**) maximum air temperature (This figure was created in the Python 3.9 and the Jupyter notebook programming interface).

The monthly performance of the raw and bias-corrected GCMs outputs for precipitation, maximum and minimum air temperature at six synoptic stations are depicted in Fig. 3. Given the superior performance of the bias-corrected outputs of ACCESS-CM2 (AC2), CNRM-CM6-1 (CC1), GFDL-ESM4 (GE4), MIROC-ES2L (MEL), and MRI-ESM2-0 (ME0), they were selected for further investigations of future climatic variables across the AWW. The KS of raw GCM data compared to observations was generally greater than 0.3 (Fig. 3a). The smaller the KS values, the better fitting of the GCM outputs to the observational data can be expected. Using the quantile delta mapping (QDM) method, the KS was reduced to less than 0.1 for all the GCMs (Fig. 3a). However, both raw and bias corrected GCMs did not achieve robust scores for CC and NRMSE indices, mainly due to the significant initial bias in the GCMs. Previous studies in the north of Iran and in the vicinity of the AWW also reported poor GCMs-CMIP6 performance for precipitation^61^. Application of QDM improved the GCMs performance (Fig. 3b,c). The average CC and NRMSE after bias correction improved compared to the raw GCMs, especially for C3 and C5 stations, which CC index reached near 0.5 following the bias correction using QDM, while CC for other stations also improved. For the maximum and minimum air temperature, both raw and biased-corrected outputs of GCMs performs quite well relative to the observational data, and the results obtained from biased-corrected GCMs show slightly better performance (Fig. 3d,e).

**Figure 4:**
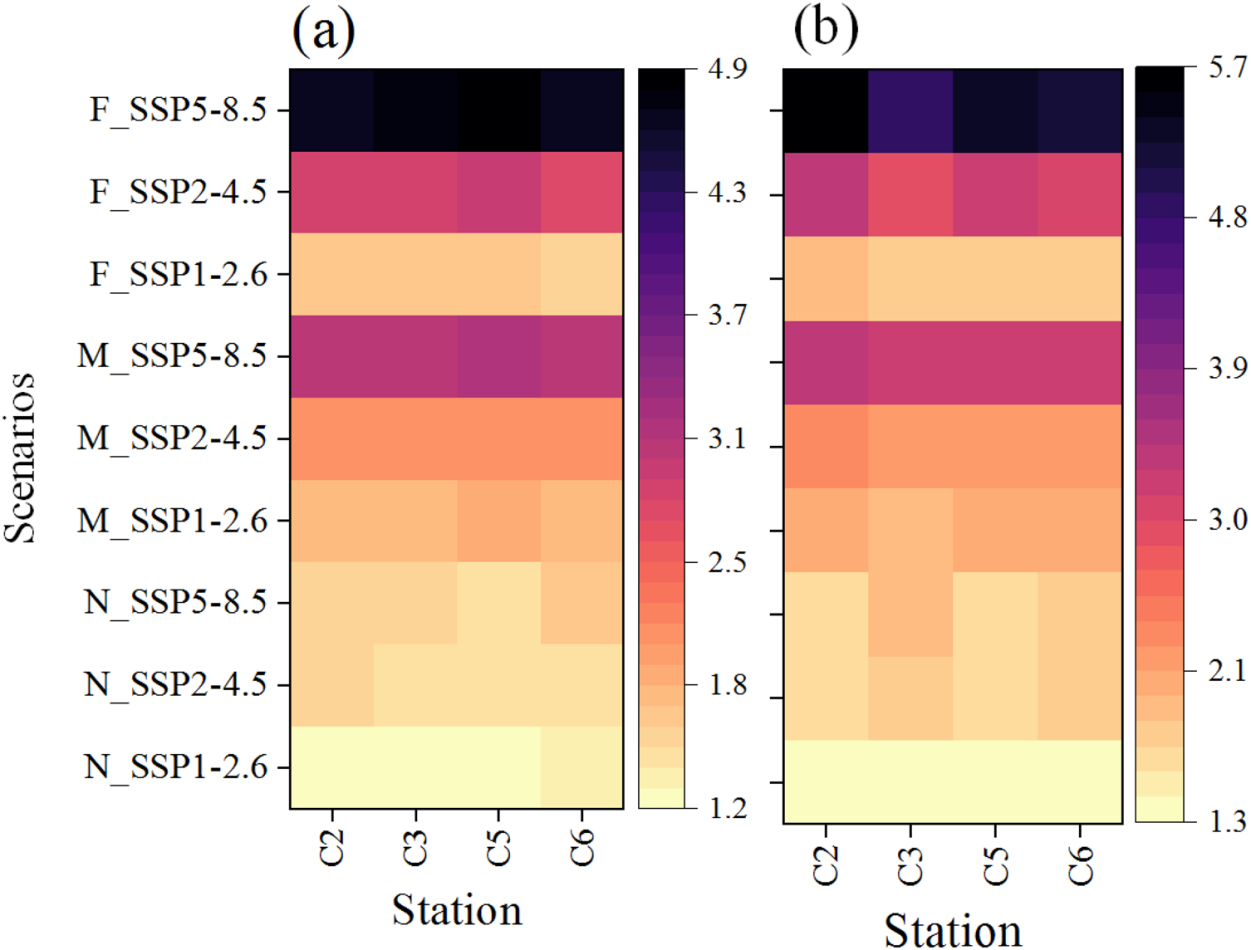
Average annual maximum and minimum air temperature changes in synoptic stations relative to the base period (1986–2020) in near (2021–2050), mid (2051–2080), and far future (2081–2100) for the mean multi-GCMs. (**a**) Minimum air temperature changes and (**b**) maximum air temperature changes*.* N: near future, M: mid future, and F: far future (This figure was created in the Python 3.9 and the Jupyter notebook programming interface).

The variations of the maximum and minimum air temperature and precipitation due to climate change effects can influence the hydrologic, ecosystem and hydraulic processes across the AWW threatening the health and functions of the Anzali wetland. The changes in the maximum and minimum air temperature are evaluated for mean multi-GCMs for SSP1-2.6, SSP2-4.5, and SSP5-8.5 in the near future (2021–2050), mid future (2051–2080), and far future (2081–2100) (Fig. 4). The projections of the mean annual maximum and minimum air temperature in the near future (i.e. 2021–2050) imply that the results are marginally sensitive to climate scenarios, similar to the findings reported by^62^. For the mid-future (i.e. 2051–2080), all climate scenarios show increased annual maximum and minimum air temperature. According to the results for SSP1-2.6 scenario, and compared to the base period, the annual maximum and minimum air temperature will increase in synoptic stations between 1.97–2.11 and 1.82–1.88 °C, respectively. Also, the annual maximum and minimum air temperature will increase in synoptic stations between 2.17–2.38 °C, 2.08–2.12 °C for SSP2-4.5, and 3.21–3.44 °C, 3.01–3.13 °C for SSP5-8.5 climate scenarios, relative to the base period. Previous studies have suggested a significant increase in the mean temperature will occur in the far future (i.e. 2081–2100), under both SSP2-4.5 and SSP5-8.5 scenarios^62,63^. The results obtained for the SSP2-4.5 indicate that the annual maximum and minimum air temperature will increase in synoptic stations between 2.95–3.41 °C and 2.76–2.93 °C, respectively, relative to the base period.The findings indicate that the most significant changes are likely to occur in the autumn and winter seasons across the AWW. The projected rise in air temperature could have severe ramifications, including the alteration of precipitation patterns within the basin, resulting in a shift from snow-rainfall to rainfall-only events. This shift could cause snow to melt during the winter season instead of spring and increase the water holding capacity, potentially leading to extreme precipitation events^60^. According to the SSP5-8.5 scenario, the annual maximum and minimum air temperature will increase in synoptic stations between 4.81–5.65 and 4.72–4.95 °C, compared to the base period in the far future, which will exert significant stress on aquatic organisms^64^. The SSP1-2.6 climate scenario, in accordance with the CMIP6 reports, implies a descending trend for the annual maximum and minimum air temperature in the far future^65^.

**Figure 5:**
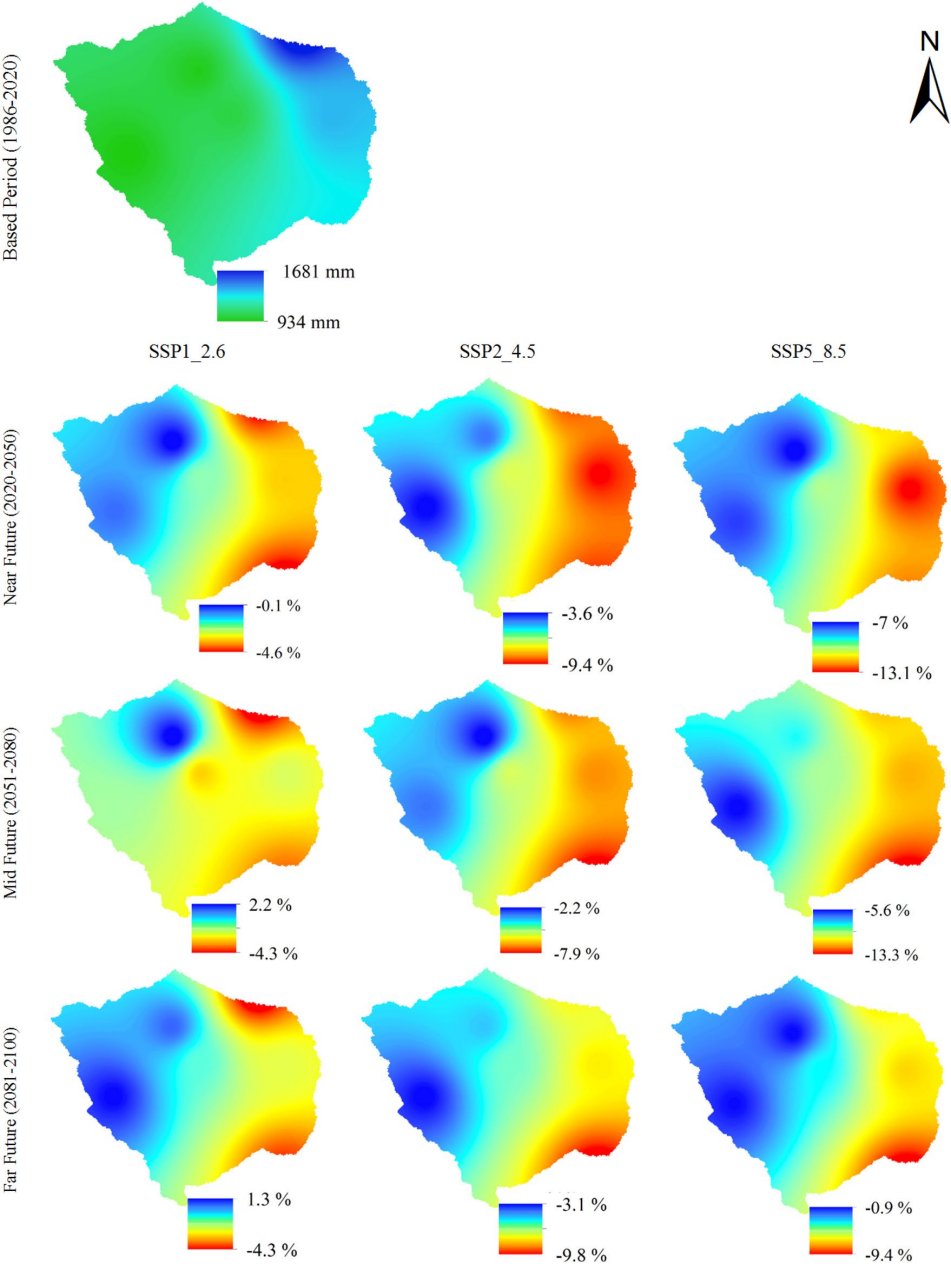
Mean annual precipitation over the basin for the base period (1986–2020), and the spatial distribution of changes in precipitation for mean multi-GCMs under SSP1-2.6, SSP2-4.5, and SSP5-8.5 in the near future (2021–250), mid future (2051–2080), and far future (2081–2100) (This figure was created in the environment of QGIS, version 3.2).

Figure 5 presents the spatial distribution of the average precipitation in the AWW projected by the mean multi-GCMs for SSP1-2.6, SSP2-4.5, and SSP5-8.5 in the near future (2021–2050), mid future (2051–2080), and far future (2081–2100), relative to the base period (1986–2020). The results highlight that east and the northeast parts of the AWW will experience more decline in the average precipitation relative to the base period. The near future changes in precipitation, relative to the base period, is expected to vary from − 0.1 to − 4.6%, − 3.6 to − 9.4%, and − 7 to − 13.1% for SSP1-2.6, SSP2-4.5, and SSP5-8.5 scenarios, respectively. The mid future changes in precipitation, compared to the base period, is projected to vary from 2.2 to − 4.3%, − 2.2 to − 7.9%, and − 5.6 to − 13.3% for SSP1-2.6, SSP2-4.5, and SSP5-8.5 scenarios, respectively. It is likely that under SSP1-2.6 scenario, the west part of the AWW would experience an increase in precipitation in the near future.

The far future changes in precipitation, relative to the base period, is projected to vary from 1.3 to − 4.3%, − 3.1 to − 9.8%, and − 0.9 to − 9.4% for SSP1-2.6, SSP2-4.5, and SSP5-8.5 scenarios, respectively. Figure 5 illustrates climate change influence on the decline of precipitation across the AWW, relative to the base period. The results show that under climate scenarios SSP2-4.5 and SSP5-8.5 and intensified decline in participation can be expected, confirming the findings of^59^. The previous results under representative concentration pathway (RCP) scenarios of RCP2.6, RCP4.5, and RCP8.5 have demonstrated a decreasing trend in precipitation. This study, compared to the previous research, uses more recent data to conduct a comprehensive assessment of climate change impacts on the AWW. Further, the CMIP6 models is used instead of the CMIP5, to better represent the watershed's future climate conditions^21^.

The spatial distributions of mean seasonal changes in precipitation are demonstrated in Supplementary Fig. S1a–d. The projection shows that in the spring, for all scenarios and time steps, the average amount of precipitation is likely to increase relative to the base period. In contrast, in the summer, when a considerable amount of the water is used for irrigation from local rivers, the model projected a damping reduction relative to the base period. Although this decline varies between scenarios and time steps, a significant decline would probably happen for SSP5-8.5 scenario and in the far future. In fall, the average precipitation in the watershed will decrease relative to the base period; especially the SSP2-4.5 and SPP5-8.5 scenarios show more significant decline compared to SSP1-2.6. Projections for the winter period show that the south and the southwest parts of the AWW will experience an increase in the average precipitation.

### Calibration and validation of the SWAT model

A sensitivity analysis is carried out to examine and evaluate effects of parameters on the model simulation of streamflow and sediment load. Global sensitivity analysis was employed in SWAT-CUP, and the most sensitive parameters influencing the streamflow are determined based on the highest *t*-stat and lowest *P*-values (Table 2). The same procedure is adopted for sensitivity analysis and calibration of the model for sediment yield. These parameters were used in previous studies of the AWW^2,59^, and in similar studies in other parts of the world^54,66,67^. The values of the parameters employed for the calibration of streamflow and sediment yield at the upstream and downstream of each hydrometric station of the AWW are presented in Table S2. The sensitivity analysis indicates CN2 is the most sensitive parameter, which is highly associated with land cover. The calibration results illustrate that upstream, which is mainly covered by forest, CN2 value is decreased, but in contrast, downstream, where rice irrigation occurs, CN2 value increases, which agrees with the previous study in the AWW^2^. The calibration results show that the forest areas, with higher water holding capacity, produce less surface runoff. Based on the calibration results, USLE_C values for forest, orchard, pasture, bare land, and rice classes were set to 0.0015, 0.005, 0.009, 0.31, and 0.008, respectively, demonstrating how LULC change influence the sediment yield across the AWW. This is specifically more pronounced when forest areas convert to pasture and bare land. It should be noted that the fitted value of sensitive parameters to streamflow and sediment yield are presented in Table 2.

**Table 2:** Summary of the most sensitive parameters influencing the streamflow and sediment yield in the Anzali wetland watershed*.*

Parameters for streamflow	Description	*t*-stat	*P*-values
CN2.mgt	Initial SCS runoff curve number for moisture condition II	12.08	< 0.001
DEP_IMP.hru	Depth to impervious layer for modeling perched water tables (mm)	− 8.83	< 0.001
SLSOIL.hru	Slope length for lateral subsurface flow (m)	− 8.36	< 0.001
ALPHA_BNK.rte	Base flow alpha factor for bank storage (days)	6.96	< 0.001
SOL_AWC(..).sol	Available water capacity of the soil layer (mm H_2_O/mm soil)	− 6.50	< 0.001
GWQMN.gw	Threshold depth of water in the shallow aquifer Required for return flow to occur (mm H2O)	5.65	< 0.001
SOL_BD(..).sol	Moist bulk density (Mg/m^3^ or g/cm^3^)	4.90	< 0.001
SOL_K(..).sol	Saturated hydraulic conductivity (mm/h)	4.57	< 0.001
CH_K1.sub	Effective hydraulic conductivity in tributary channel alluvium (mm/h)	4.27	< 0.001
Parameters for sediment yield			
PRF_BSN.bsn	Peak rate adjustment factor for sediment routing in the main channel	− 13.82	< 0.001
USLE_P.mgt	USLE equation support practice factor	− 9.10	< 0.001
CH_COV2.rte	the channel cover factor	− 8.87	< 0.001
USLE_K(..).sol	USLE equation soil erodibility (K) factor	− 8.87	< 0.001
USLE_C.crop.dat	Minimum value of USLE C factor for water erosion applicable to the land cover/plant	3.67	< 0.001

Monthly calibration and validation results for streamflow and sediment load at each station are determined based on NSE, R^2^, R-*factor*, and P-*factor* (Table 3). Calibration and validation results for streamflow showed that the overall model performance was satisfactory (NSE ≥ 0.5, R^2^ ≥ 0.6)^54–56^, except for the station five (R-*factor* 71%, and P-*factor* 70%) where observation data in the calibration and validation period fell in the 995PPU. It should be noted that the AWW is a complex watershed with diverse and broad LULC. To this date, no information exists on the withdrawal amount of water by deviation channels for irrigation and water consumption of the adjacent villages, and these uncertainties can affect the model’s performance. The overall performance of the model during calibration and validation is in the similar range as the previous study^2^. While in the calibration and validation process, the S7 river, which provides approximately 30% of the wetland's inflow, performed better than the previous study^2^ with perfect NSE (0.86) and R^2^ (0.86) values, which causes a better representation of future conditions of the Anzali wetland inflow under climate and LULC change. It should be noted that PLAPS ranges were adjusted from − 63 to − 200 mm, and TLAPS ranges from − 2.3 to − 4.5 °C/1 km across the AWW.

**Table 3:** Statistical performance of the SWAT model for calibration and validation periods.

Monitoring gauge	Calibration period (1989–2012)				Validation period (2013–2020)			
	R-*factor*	P-*factor*	NSE	R^2^	R-*factor*	P-*factor*	NSE	R^2^
Streamflow								
S1	1.26	0.84	0.67	0.69	1.09	0.81	0.56	0.63
S2	0.88	0.71	0.61	0.62	1.01	0.73	0.54	0.64
S3	1.11	0.76	0.78	0.78	1.15	0.77	0.65	0.72
S4	1.07	0.73	0.71	0.72	1.06	0.83	0.65	0.74
S5	1.05	0.71	0.58	0.59	1.08	0.70	0.45	0.50
S6	0.88	0.64	0.67	0.67	0.84	0.68	0.55	0.56
S7	0.58	0.70	0.86	0.86	0.88	0.68	0.74	0.75
S8	0.80	0.73	0.64	0.65	1.14	0.86	0.60	0.72
Maximum	1.26	0.84	0.86	0.86	1.15	0.86	0.74	0.75
Minimum	0.58	0.64	0.58	0.59	0.84	0.68	0.45	0.50
Average	0.95	0.73	0.69	0.70	1.03	0.76	0.59	0.66
Sediment load								
S1	0.60	0.64	0.60	0.60	0.74	0.71	0.57	0.64
S2	0.43	0.84	0.57	0.60	0.66	0.65	0.57	0.58
S3	0.68	0.85	0.65	0.68	1.06	0.77	0.59	0.64
S4	1.05	0.78	0.64	0.76	1.34	0.76	0.55	0.67
S5	0.56	0.76	0.48	0.53	0.83	0.61	0.41	0.44
S6	0.93	0.81	0.60	0.61	0.86	0.76	0.57	0.57
S7	0.7	0.85	0.79	0.83	0.63	0.77	0.65	0.68
S8	0.53	0.81	0.57	0.65	0.88	0.95	0.81	0.85
Maximum	1.05	0.85	0.79	0.83	1.34	0.95	0.81	0.85
Minimum	0.43	0.64	0.48	0.53	0.63	0.61	0.41	0.44
Average	0.69	0.79	0.61	0.66	0.88	0.75	0.59	0.63

Monthly calibration and validation were carried out for sediment load with the sediment rating curve method. R^2^ was used to determine the performance of the sediment rating curve method. R^2^ was 0.79, 0.62, 0.6, 0.74, 0.74, 0.77, 0.69, and 0.78 for S1, S2, S3, S4, S5, S6, S7, and S8, respectively. Arnold et al.^54^ highlighted that the model's performance for sediment load calibration depends on how streamflow is calibrated. The average NSE of sediment calibration (0.61) and validation (0.59) is similar to previous studies conducted for different time periods^2^. The model’s result showed that the sediment load model acted not very well in S5 and was returned to the flow calibration of their stations. For instance, streamflow performed perfectly in S7 and S4 stations, and sediment load calibration acted well. However, in S5, streamflow performed not very well, and sediment load showed the same result, but the R-*factor* (≤ 2 or 3) and P-*factor* (≥ 0.5 or 0.4) for sediment load indicated that the model performed well^56^. Figure S2 provides additional information on the hydrograph performance of the SWAT model for streamflow and sediment load at eight stations, both during the calibration and validation period.

### Climate and LULC change impacts on streamflow and sediment load

**Figure 6:**
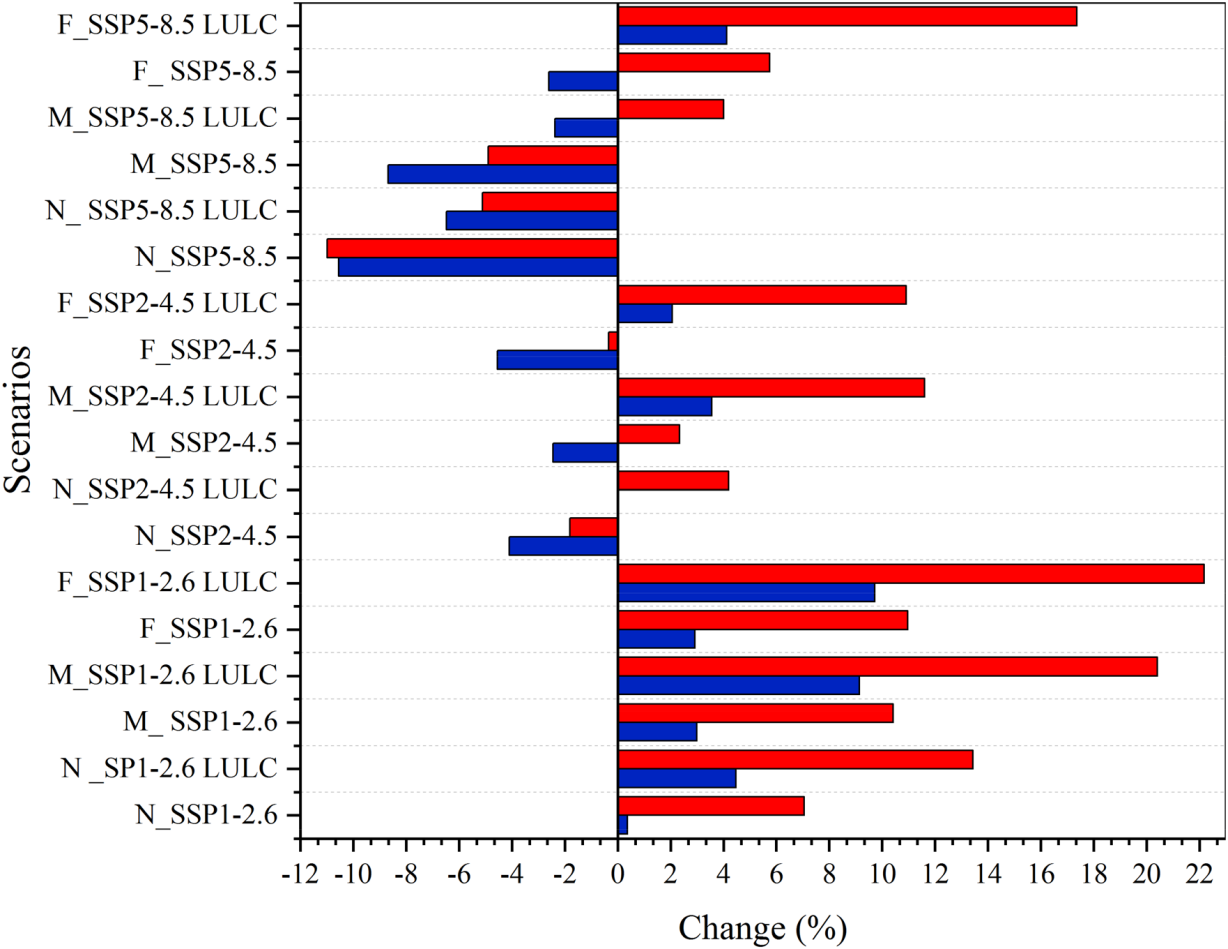
Variation in average streamflow and sediment load under mean multi-GCMs and land use land cover (LULC) changes for different scenarios in different time steps relative to the base period (1989–2020). N: Near future (2021–2050), M: mid future (2051–2080), and F: far future (2081–2100)*.* Blue bar: streamflow and red bar: sediment load (This figure was created in the Python 3.9 and the Jupyter notebook programming interface).

Mean multi-GCMs cumulative changes in streamflow and sediment load for the Anzali wetland under the SSP1-2.6, SSP2-4.5, and SSP5-8.5 climate scenarios are presented in Fig. 6. The results showed in Fig. 6 considers the simultaneous impacts of climate and LULC changes. The precipitation and air temperature will not significantly alter under SSP1-2.6 (Figs. 4 and 5), especially in the south of the AWW, where rivers emanate. Hence, no significant change is observed for the projected streamflow, compared to the base period (1989–2020), whilst sediment load increases. For the far future (2081–2100) results, a meaningful increase in streamflow (up to 9.7%) and sediment load (up to 22.2%) is observed relative to the base period when the impacts of LULC changes is incorporated in the SSP1-2.6 scenario, which is mainly due to a significant increase in urbanization and deforestation in the AWW.

For the SSP2-4.5 scenario, the projected results under the influence of climate change show a decrease in precipitation, and an increase in Tas_max_ and Tas_min_, relative to the base period, leading to a decrease in streamflow and sediment load up to 4.6% and 0.4%, respectively, in the far future. In addition, high precipitation during the winter, increase sediment load when vegetation cover is low in the AWW, which will consequently lead to a mean sediment load increase (up to 5.7%), despite streamflow decrease (see Fig. 6). Inclusion of the LULC changes in the modelling increase the streamflow up to 2% and 4.1% for SSP2-4.5 and SSP5-8.5, respectively. Similarly, sediment load increases up to 10.9% and 17.4% for SSP2-4.5 and SSP5-8.5 scenarios, respectively. These findings ascertain the sensitivity of streamflow, and specifically, sediment load to deforestation and urbanization. The sediment load increases more than the streamflow as the local anthropogenic activities intensify the erodibility factor, even if a decline in the precipitation is observed. The findings indicate that a reduction in the rate of deforestation, which serves as a natural sediment trap^60,68^, can effectively safeguard the Anzali wetland area against sedimentation. Such sedimentation has the potential to fill up the wetland, thereby disrupting its ecosystem health^69^ and function in the AWW region.

**Figure 7:**
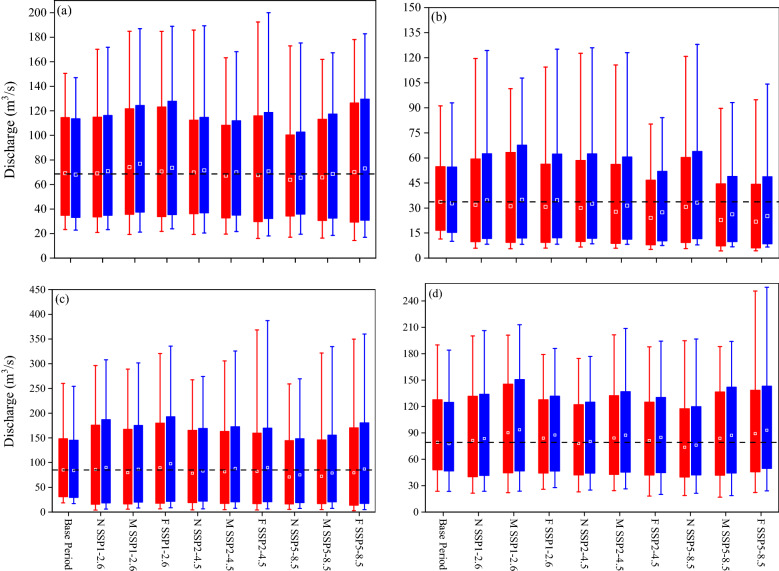
Box plot of the seasonal streamflow under multi-GCMs and land use land cover (LULC) changes for different scenarios to compare the variation among base period (1989–2020), near future (2021–2050), mid future (2051–2080), and far future (2081–2100) in (**a**) spring, (**b**) summer, (**c**) fall, and (**d**) winter. N: near future, M: mid future, and F: far future. Red box: climate change and blue box: climate and LULC changes. Dashed line indicates the average streamflow to the Anzali wetland during the base period (This figure was created in the Python 3.9 and the Jupyter notebook programming interface).

**Figure 8:**
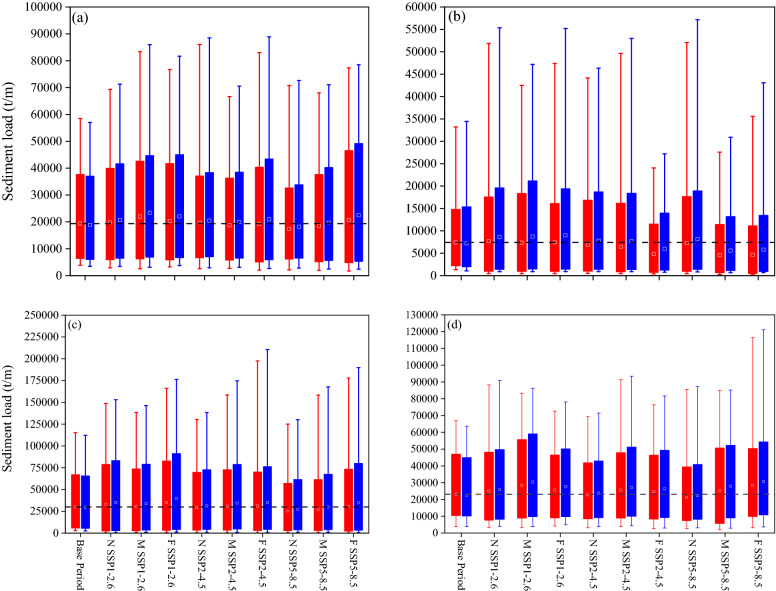
Box plot of the seasonal sediment load projected under the multi-GCMs and land use land cover (LULC) changes for different scenarios to compare the variation among base period (1989–2020), near future (2021–2050), mid future (2051–2080) and far future (2081–2100) in (**a**) spring, (**b**) summer, (**c**) fall, and (**d**) winter. N: near future, M: mid future, and F: far future. Red box: climate change and blue box: climate and LULC changes. Dashed line indicates the average sediment load to the Anzali wetland during the base period (This figure was created in the Python 3.9 and the Jupyter notebook programming interface).

Seasonal streamflow and sediment load to the wetland projected by multi-GCMs are shown in Figs. 7 and 8, respectively. When the impacts of climate and LULC changes are considered simultaneously, the projected streamflow and sediment load increased, compared to the results obtained for climate change impacts only (Figs. 7 and 8). This signifies the influence of the future urbanization and deforestation projected by the LCM, which becomes more pronounced for the far future projections across the watershed (Table 1). Urbanization increases peak flows across the AWW due to a reduction in infiltration capacity^30,70^. Deforestation reduces the storage capacity of the AWW resulting in intensified sediment yields and surface runoff in the watershed^2,14^.

Multi-GCMs suggest seasonal variations of streamflow input to the wetland, with a minimum flow in the summer when both Tas_max_ and Tas_min_ are maximum, precipitation is significantly reduced (Fig. S1b), and irrigation water demand is maximum across the AWW. The decline (relative to base period) in summer low streamflow (10% minimum quantile) to the wetland under the combined impacts of climate and LULC changes, and for the most descending time step, is around 30%, 33%, and 48% under the SSP1-2.6, SSP2-4.5, and SSP5-8.5 scenarios, respectively. When excluding the influence of LULC changes in the modelling, summer low streamflow (10% minimum quantile) to the wetland further reduces compared to the base period (i.e. 49%, 55%, and 65% for the SSP1-2.6, SSP2-4.5, and SSP5-8.5 scenarios, respectively). The results for the impact of LULC on damping reduction of low streamflow are in agreement with the existing studies that suggest deforestation increase the minimum streamflow^30,71^. The projected trend of declining mean low streamflow during the fall period has significant implications for the protection of the wetland's biodiversity, biogeochemical processes, and water quality^69^.

With respect to the high streamflow and sediment load (i.e. 10% maximum quantile), the AWW experiences high extreme turbid events with large sediment load due to more extreme precipitation under the impact of sole climate change during the spring, fall, and winter (Figs. 7 and 8). Inclusion of the LULC in the simulations further increase the high extreme streamflow and sediment load due to extensive deforestation and urbanization in the AWW. The climate change increases the projected high streamflow and sediment load, in the most ascending time step, by around 11% and 27% (in spring), 25% and 40% (in fall), and 16% and 29% (in winter) under the SSP1-2.6, SSP2-4.5, and SSP5-8.5 scenarios, respectively. The simulations show the inclusion of LULC changes in the model further increases the projected high streamflow and sediment load to 16% and 34% (in spring), 32% and 51% (in fall), and 20% and 37% (in winter) under the SSP1-2.6, SSP2-4.5, and SSP5-8.5 scenarios, respectively (Figs. 7 and 8). It can be concluded that the sediment load is more sensitive to the LULC changes than the streamflow in the AWW.

Overall, the findings of this study suggest that the forest area, mainly located on high slope zones, significantly prevents sediment load and high streamflow production in the sub-basins of the AWW. Projected total sediment input to the Anzali wetland reach 20.94, 19.19, and 18.30 million tons by 2100 (from 2021) under SSP1-2.6, SSP2-4.5 and SSP5-8.5 scenarios, respectively. For the combined effects of climate change and LULC changes, the sediment loads increase to 22.66, 20.83, and 19.93 million tons under SSP1-2.6, SSP2-4.5 and SSP5-8.5 scenarios, respectively. The large sediment inputs projected will degrade the Anzali wetland ecosystem^69^. In recent decades, water depth has been decreasing in various areas of the Anzali wetland^1^ primarily due to the substantial sediment loads that have been deposited. This process could be exacerbated in the future due to potential climate and land use changes. Furthermore, the projected decrease in streamflow in both the SSP2-4.5 and SSP5-8.5 scenarios compared to the base period, along with a projected decline in the water level of the Caspian Sea^72–74^, could lead to further reduction of the Anzali wetland's size. This poses a significant threat to the wetland's biodiversity, water quality, and biogeochemical processes^1^, and result in resigning the wetland from the Montreux record list and the Ramsar Convention on Wetlands of International Importance. Additionally, the sediment loads can intensify the transport of heavy metals^75–78^, nutrients, and other pollutants to the wetland^79^, with adverse implications for the ecosystem, aquatic life, food-web chains, and water quality^80–82^.

## Conclusions

This study investigated the effects of the climate and LULC changes on streamflow and sediment load to the Anzali wetland using SWAT model. Different land classes were identified and quantified across the AWW. For the first time, this study distinguished the rice class from the rain-fed orchard class, and bare land from pasture across the AWW, which can highly influence the water budget in a watershed scale. In addition, enhanced representation of the Anzali watershed conditions and reduced modelling uncertainty was presented using the CMIP6 multi-model, instead of CMIP5 adopted in the previous studies. Combined effects of climate change and LULC changes highlighted that deforestation and urbanizations will intensify streamflow and sediment load across the AWW. The results showed that despite the primary role of climate change in determining changes in streamflow and sediment load, LULC changes effects were also significantly influencing the hydrological processes across the AWW. Therefore, the ecosystem health and functions of the Anzali wetland is endangered under the future climate scenarios, and will be further deteriorated if the climate impacts are combined with the already projected changes in the LULC in the watershed. It is important to acknowledge that the sediment concentration data used in this study was based on monthly measurements rather than daily gauges in the AWW. To estimate sediment loads, the sediment rating curve method was utilized. Furthermore, the LCM simulations did not account for changes in water class due to external factors such as fluctuations in precipitation, temperature, and the Caspian Sea mean water line, which can introduce additional uncertainty in the presented results.

## Supplementary Information

Supplementary Information.

## Data Availability

The datasets analyzed during the current study are available from the corresponding author on reasonable request.
