# Supplementary material for: Modelling impacts of climate change and anthropogenic activities on inflows and sediment loads of wetlands: case study of the Anzali wetland
Source: Sci Rep. 2023 Apr 3;13:5399. doi: 10.1038/s41598-023-32343-8 (PMC10070450; doi:10.1038/s41598-023-32343-8)
Supplement: Supplementary file 1 — Supplementary Information. [file 41598_2023_32343_MOESM1_ESM.docx]

**Modelling impacts of climate change and anthropogenic activities on inflows and sediment loads of wetlands: Case study of the Anzali wetland**

Mehran Mahdian^1^, Majid Hosseinzadeh^1,*^, Seyed Mostafa Siadatmousavi^1^, Zohreh Chalipa^1^, Majid Delavar^2^, Ming Guo^3^, Soroush Abolfathi^3^, Roohollah Noori^4,5^

^1^School of Civil Engineering, Iran University of Science and Technology, Narmak, Tehran 1684613114, Iran.

^2^Department of Water Resources Engineering, Tarbiat Modares University, Tehran 14115-111 Iran.

^3^School of Engineering, University of Warwick, Coventry CV4 7AL, UK.

^4^Graduate Faculty of Environment, University of Tehran, Tehran 1417853111, Iran.

^5^Faculty of Governance, University of Tehran, Tehran 1439814151, Iran.

^*^[hosseinzadeh_m@iust.ac.ir](mailto:hosseinzadeh_m@iust.ac.ir) (M.H), ORCID: <https://orcid.org/0000-0003-0542-8869>

Supplementary

**Text S1.** LULC change has some driving factors categorized as natural and anthropogenic activity. In our study, the statistic variables of DEM, slope, distance from the roads, closeness to rivers, distance from the city, distance from the wetland, and evidence likelihood were chosen as natural and anthropogenic driving factors as suggested by previous studies. Each variable was selected for the following reasons.

- The elevation change determines how appropriate an area is for agriculture.
- The slope has a significant impact on deforestation.
- The distance from the city is important because the LULC transformation rate is higher for land categories close to the residential areas.
- The LULC transformation rate is higher for areas close to the road.
- Closeness to the river is important for agriculture.
- Distance from the wetland should be considered because the wetland is a protected but fertile region.
- The evidence likelihood is an empirical possibility of change in the LULC classes between an earlier and a later map.

| Model Name | Country | Resolution  (latitude × longitude) | Institute |
| --- | --- | --- | --- |
| ACCESS-CM2 | Australia | 1.25° × 1.87° | Commonwealth Scientific and Indus-trial Research Organisation (CSIRO), Australian Research Council Centre of Excellence for Climate System Science (ARCCSS), and Bureau of  Meteorology |
| CanESM5 | Canada | 2.79° × 2.81° | Canadian Centre for Climate Modeling and Analysis |
| CMCC-ESM2 | Italy | 0.94° × 1.25° | Euro-Mediterranean Centre on Climate Change |
| CNRM-CM6-1 | France | 1.4° × 1.4° | National Center of Meteorological Research |
| FGOALS-g3 | China | 2° × 2° | LASG, Institute of Atmospheric Physics, Chinese Academy of Sciences |
| GFDL-ESM4 | USA | 1° × 1.25° | NOAA Geophysical Fluid Dynamics Laboratory |
| HadGEM3-GC31-LL | UK | 1.25° × 1.88° | Met Office Hadley Centre |
| IPSL-CM6A-LR | France | 1.25° × 2.5° | Institute Pierre Simon Laplace |
| MIROC-ES2L | Japan | 2.79° × 2.81° | Japan Agency for Marine-Earth Science and10 Technology |
| MPI-ESM1-2-LR | Germany | 1.86° × 1.87° | Max Planck Institute for Meteorology |
| MRI-ESM2-0 | Japan | 1.12° × 1.12° | Meteorological Research Institute |
| NorESM2-LM | Norway | 1.89° × 2.5° | Norwegian Climate Service Centre |

**Table S1**. CMIP6 models used in the research

| Parameters for streamflow | S1 | S2 | S3 | | | | S4 | | S5 | | S6 | | S7 | | S8 | |
| --- | --- | --- | --- | --- | --- | --- | --- | --- | --- | --- | --- | --- | --- | --- | --- | --- |
|  | U | D | U-Left | D-Left | U-Right | D-Left | U | D | U | D | U | D | U | D | U | D |
| r^a^__CN2.mgt | -0.40 | -0.11 | -0.35 | 0.29 | -0.24 | -0.05 | -0.4 | -0.25 | -0.09 | 0.25 | -0.31 | 0.06 | -0.40 | 0.24 | -0.31 | 0.07 |
| v^b^__DEP_IMP.hru | 3894 | 1102 | 1542 | 1902 | 2226 | 2742 | 1423 | 508 | 5966 | 2630 | 2180 | 3498 | 1914 | 1990 | 1239 | 2763 |
| v__SLSOIL.hru | 15.3 | 14.3 | 150 | 140 | 49 | 145 | 35.6 | 20.5 | 8.6 | 23.8 | 0.80 | 34.8 | 23.3 | 107 | 0.40 | 75.4 |
| v__ALPHA_BNK.rte | 0.26 | 0.44 | 0.74 | 0.02 | 0.87 | 0.20 | 0.56 | 0.58 | 0.46 | 0.31 | 0.29 | 0.24 | 0.45 | 0.79 | 0.05 | 0.97 |
| r__SOL_AWC(..).sol | -0.20 | 0.10 | 0.05 | 0.51 | 0.60 | -0.70 | 0.10 | -0.30 | 0.64 | -0.70 | 0.02 | 0.70 | -0.65 | -0.3 | -0.14 | 0.18 |
| v__GWQMN.gw | 222 | 912 | 4865 | 1115 | 45 | 2055 | 686 | 4901 | 1291 | 1450 | 1099 | 2328 | 4712 | 1705 | 1816 | 2912 |
| r__SOL_BD(..).sol | -0.1 | 0.45 | 0.31 | 0.29 | 0.55 | 0.49 | -0.51 | 0.4 | 0.27 | -0.47 | 0.01 | 0.02 | 0.35 | -0.48 | 0.26 | 0.22 |
| r__SOL_K(..).sol | 0.18 | -0.1 | -0.23 | -0.5 | 0.02 | -0.18 | 0.31 | 0.37 | 0.13 | -0.32 | 0.03 | 0.02 | 0.08 | 0.25 | 0.35 | -0.25 |
| v__CH_K1.sub | 48.3 | 63 | 10.3 | 104.8 | 76.6 | 66.4 | 88.9 | 11 | 119.8 | 99.8 | 92.8 | 180.1 | 6.5 | 67.3 | 89.6 | 81.6 |
| Parameters for sediment yield |  |  |  |  |  |  |  |  |  |  |  |  |  |  |  |  |
| v__PRF_BSN.bsn | 0.88 | | | | | | | | | | | | | | | |
| r__USLE_P.mgt | -0.5 | -0.55 | -0.65 | -0.40 | -0.60 | -0.35 | -0.55 | -0.50 | -0.40 | -0.35 | -0.20 | -0.35 | -0.40 | -0.30 | -0.40 | -0.50 |
| v__CH_COV2.rte | 0.85 | 0.65 | 0.61 | 0.64 | 0.46 | 0.20 | 0.25 | 0.19 | 0.30 | 0.20 | 0.43 | 0.27 | 0.55 | 0.25 | 0.41 | 0.20 |
| r__USLE__K(..).sol | -0.50 | -0.50 | 0.44 | 0.30 | 0.48 | 0.40 | -0.50 | 0.40 | 0.25 | 0.17 | 0.25 | -0.30 | 0.20 | 0.50 | 0.42 | 0.32 |

**Table S2**. The fitted values of parameters employed for the calibration of streamflow and sediment yield at the upstream and downstream of each hydrometric station.


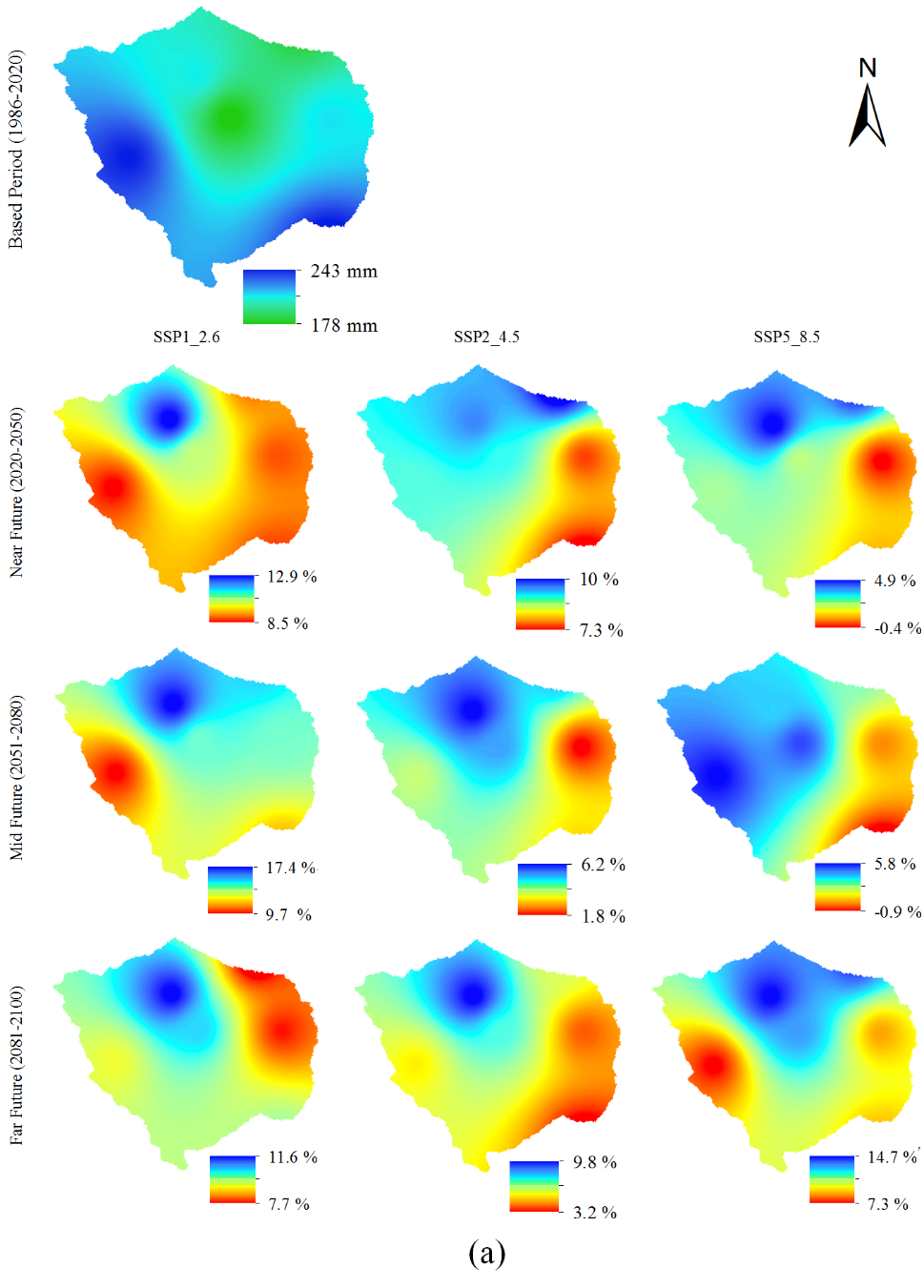


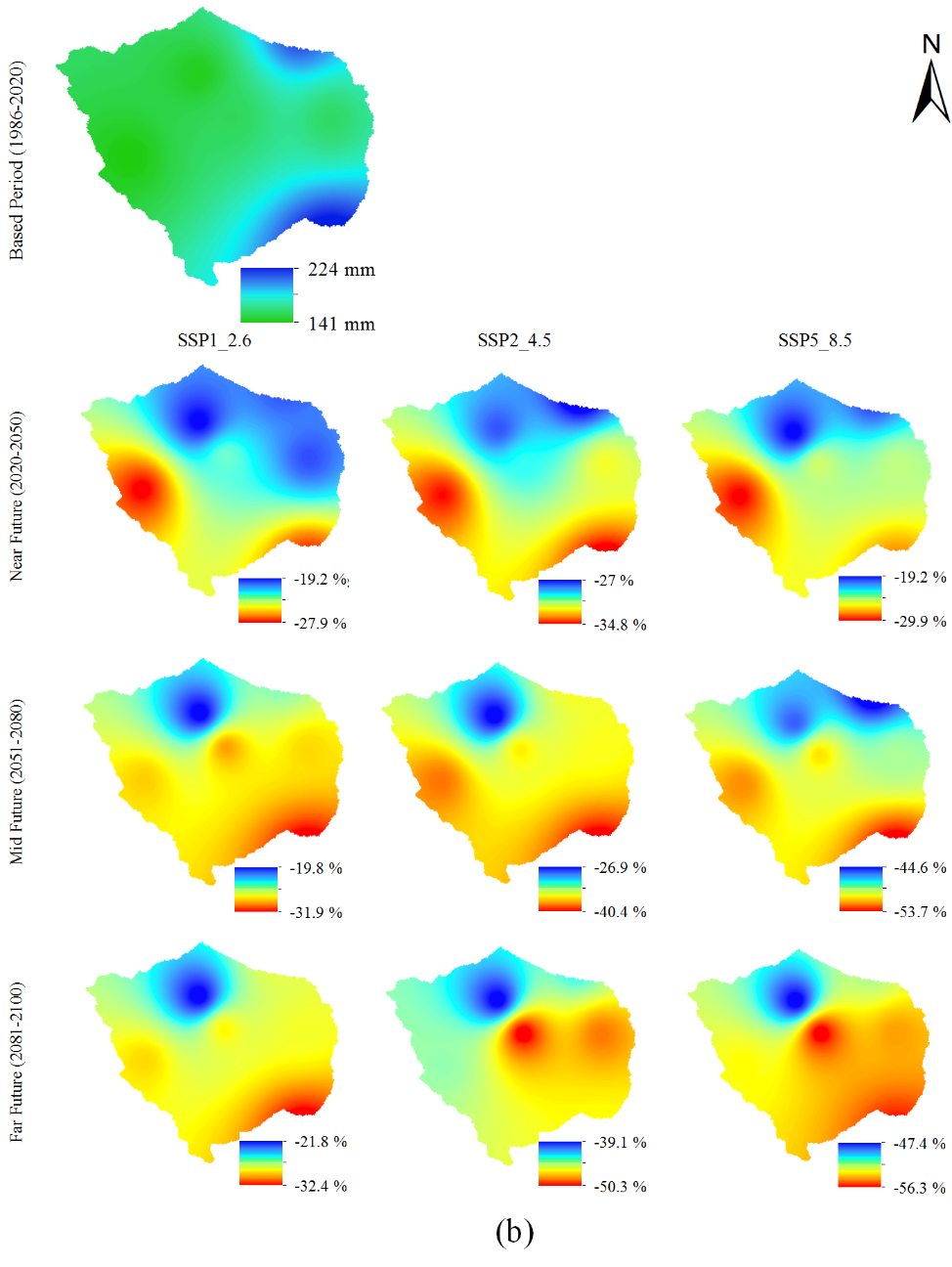


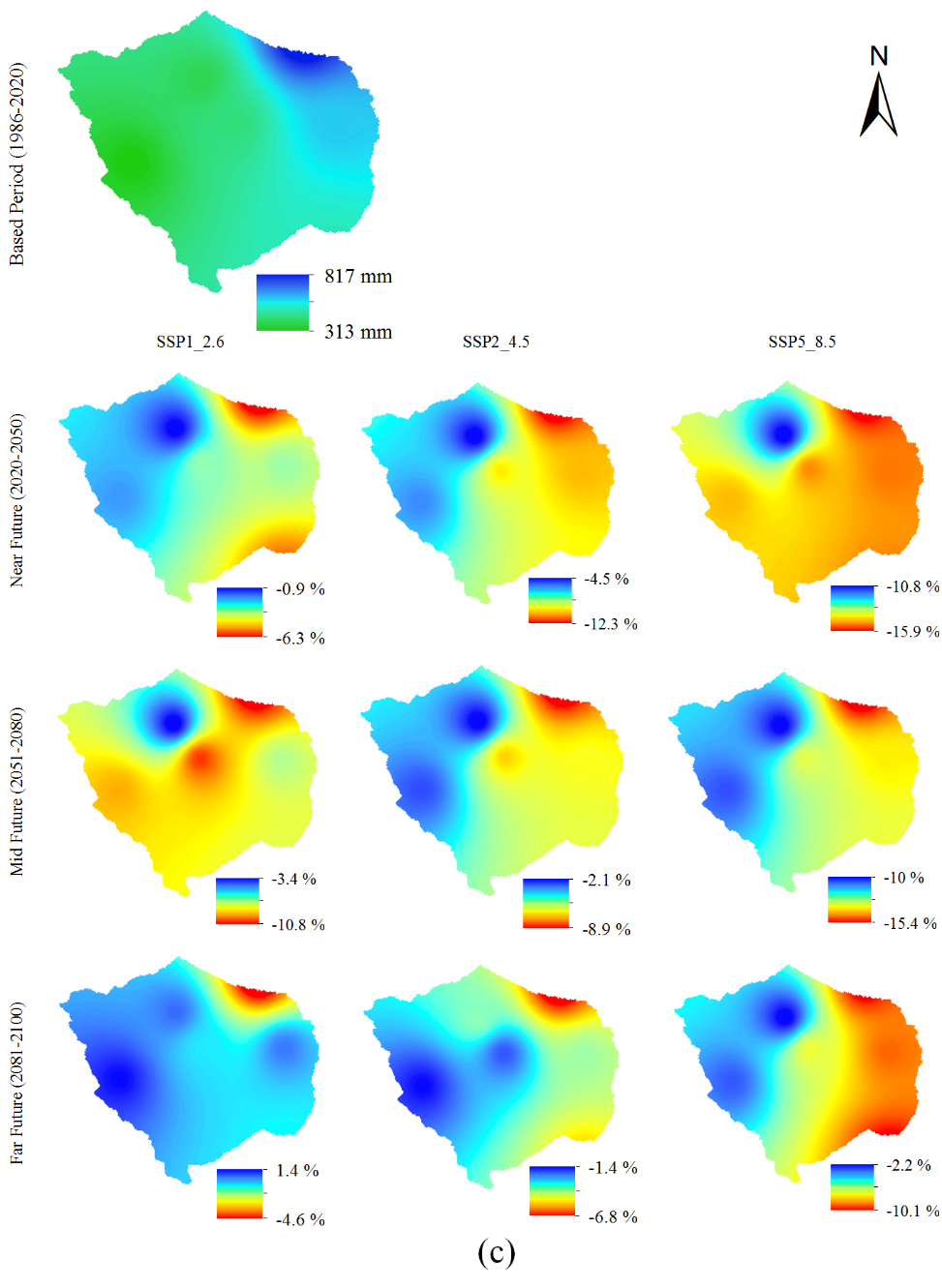
**
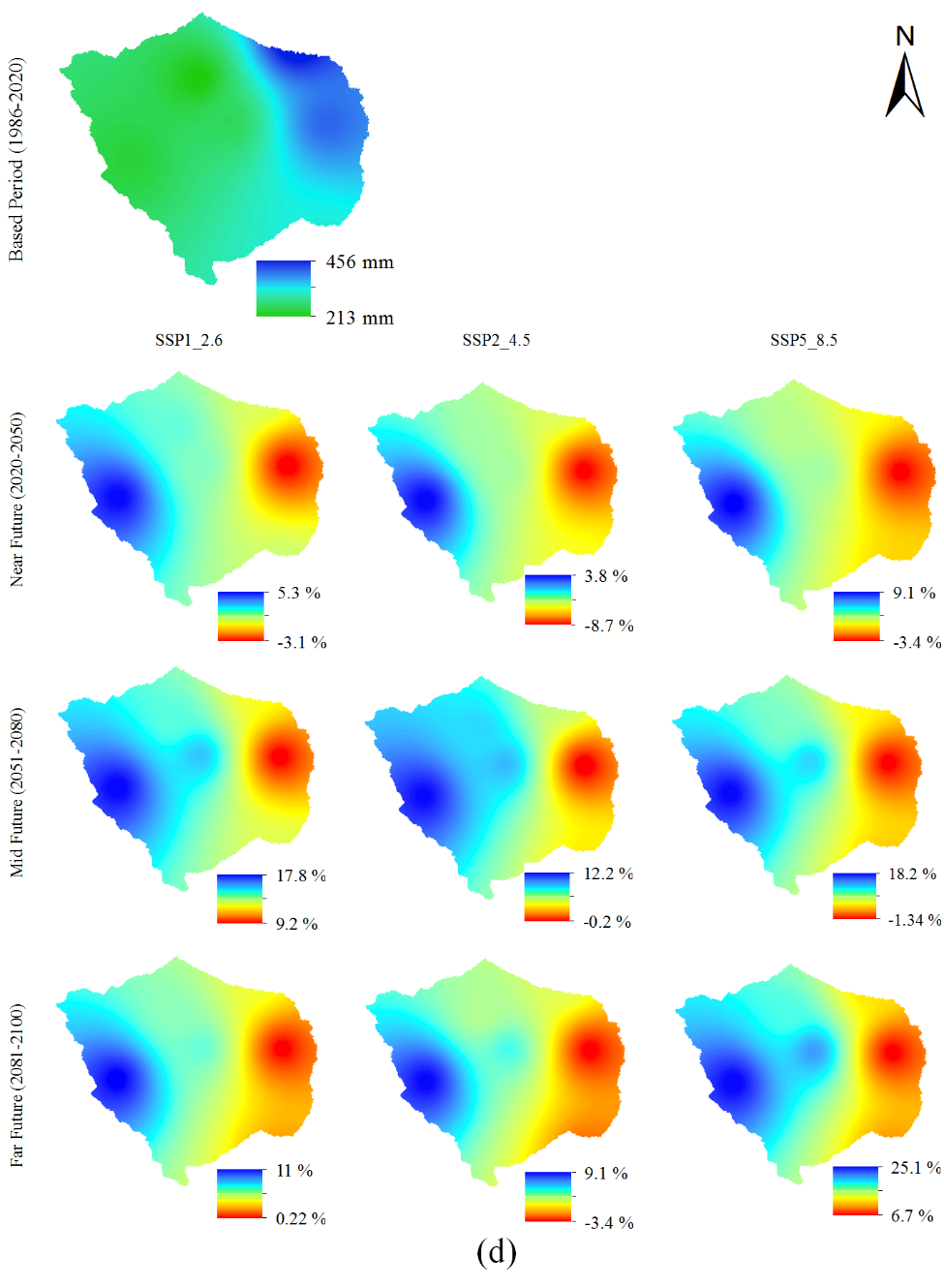
Figure S1a-S1d.** Mean seasonal precipitation over the watershed for the base period (1986-2020) and the spatial distribution of change of precipitation for mean multi-GCMs under SSP1-2.6, SSP2-4.5, and SSP5-8.5 in the near future (2021-2050), mid future (2051-2080), and far future (2081-2100). (a) spring, (b) summer, (c) fall, and (d) winter. (This figure was created in the environment of QGIS, version *3.2*).


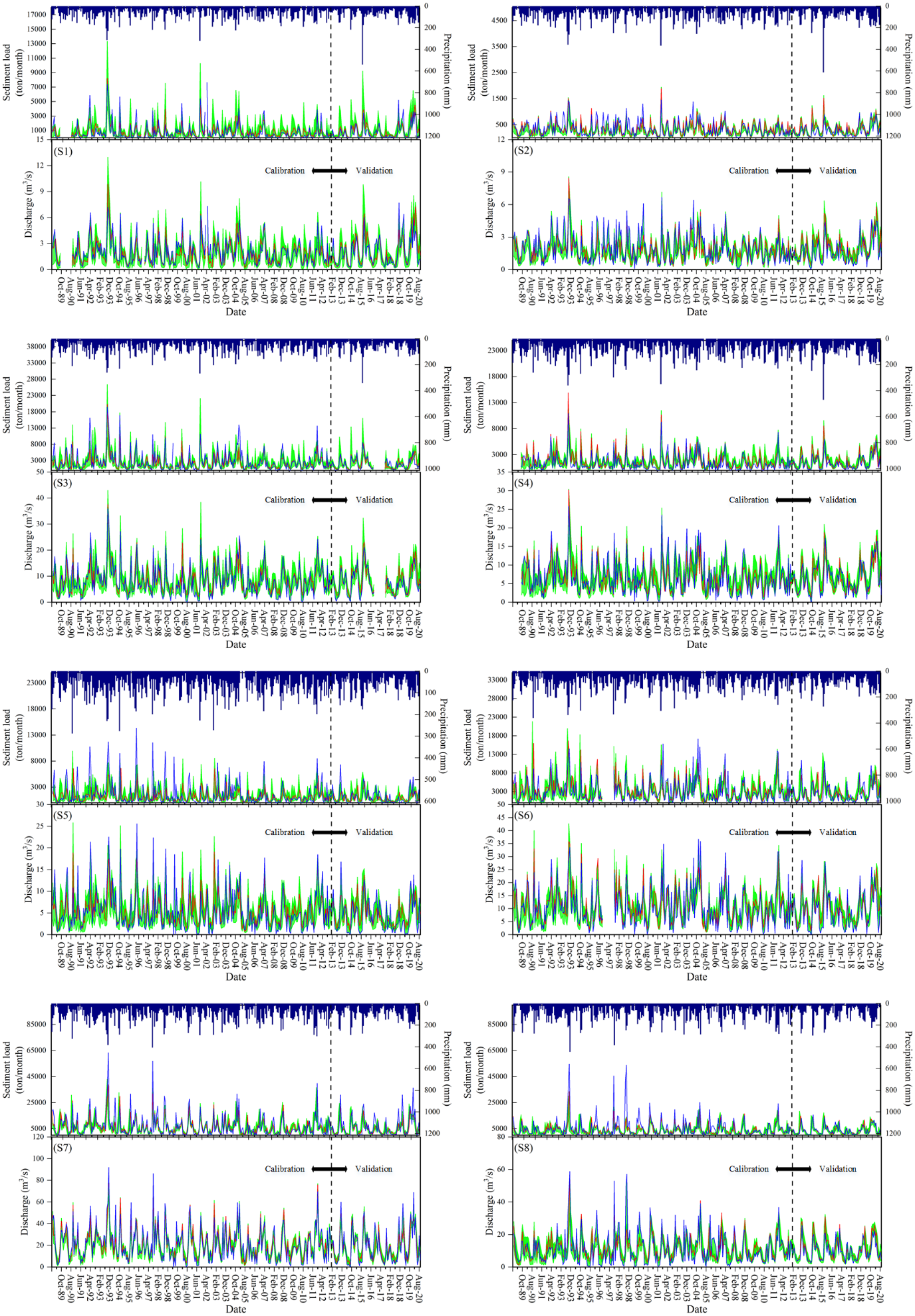


**Figure S2**. Time series of simulated monthly precipitation, stramflow and sediment load in both calibration and validation of SWAT model versue the observational once at stations S1 to S8 during the study base period (1989-2020) , and best-simulated streamflow and sediment load at the outlets of rivers of S1, S2, S3, S4, S5, S6, S7, and S8 in the calibration (1989-2012) and validation (2013-2020) period with SWAT model.. Blue and red lines represent observation and model best performance, respectively. The green band illustrates the 95 PPU for streamflow and sediment load. The dark-blue bars show the weighted average precipitation in the sub-basins of each station during the base period (1989-2020) (This figure was created in the Python 3.9 and the Jupyter notebook programming interface).
